# Establishment of in-hospital clinical network for patients with neurofibromatosis type 1 in Nagoya University Hospital

**DOI:** 10.1038/s41598-021-91345-6

**Published:** 2021-06-07

**Authors:** Yoshihiro Nishida, Kunihiro Ikuta, Atsushi Natsume, Naoko Ishihara, Maki Morikawa, Hiroyuki Kidokoro, Yukako Muramatsu, Norie Nonobe, Kanako Ishizuka, Takuya Takeichi, Miki Kanbe, Seiji Mizuno, Shiro Imagama, Norio Ozaki

**Affiliations:** 1 Department of Rehabilitation Medicine, Nagoya University Hospital, 65 Tsurumai, Showa, Nagoya, Aichi 466-8550 Japan; 2 Department of Orthopedic Surgery, Nagoya University Graduate School of Medicine, 65 Tsurumai, Showa, Nagoya, Aichi 466-8550 Japan; 3 Department of Neurosurgery, Nagoya University Graduate School of Medicine, 65 Tsurumai, Showa, Nagoya, Aichi 466-8550 Japan; 4 Department of Pediatrics, Fujita Health University School of Medicine, Toyoake, Japan; 5 Medical Genome Center, Nagoya University Hospital, 65 Tsurumai, Showa, Nagoya, Aichi 466-8550 Japan; 6 Department of Pediatrics, Nagoya University Graduate School of Medicine, 65 Tsurumai, Showa, Nagoya, Aichi 466-8550 Japan; 7 Department of Ophthalmology, Nagoya University Graduate School of Medicine, 65 Tsurumai, Showa, Nagoya, Aichi 466-8550 Japan; 8 Department of Psychiatry, Nagoya University Graduate School of Medicine, 65 Tsurumai, Showa, Nagoya, Aichi 466-8550 Japan; 9 Department of Dermatology, Nagoya University Graduate School of Medicine, 65 Tsurumai, Showa, Nagoya, Aichi 466-8550 Japan; 10 Department of Plastic and Reconstructive Surgery, Nagoya University Graduate School of Medicine, 65 Tsurumai, Showa, Nagoya, Aichi 466-8550 Japan; 11 Division of Clinical Genetics, Aichi Developmental Disability Center Hospital, 713-8 Kagiya-cho, Kasugai, Aichi 480-0392 Japan

**Keywords:** Preventive medicine, Sarcoma

## Abstract

Neurofibromatosis type 1 (NF1) is a genetic multisystem disorder. Clinicians must be aware of the diverse clinical features of this disorder in order to provide optimal care for it. We have set up an NF1 in-hospital medical care network of specialists regardless of patient age, launching a multidisciplinary approach to the disease for the first time in Japan. From January 2014 to December 2020, 246 patients were enrolled in the NF1 patient list and medical records. Mean age was 26.0 years ranging from 3 months to 80 years. The number of patients was higher as age at first visit was lower. There were 107 males (41%) and 139 females. After 2011, the number of patients has increased since the year when the medical care network was started. Regarding orthopedic signs, scoliosis was present in 60 cases (26%), and bone abnormalities in the upper arm, forearm, and tibia in 8 cases (3.5%). Neurofibromas other than cutaneous neurofibromas were present in 90 cases (39%), and MPNST in 17 cases (7.4%). We launched a multidisciplinary NF1 clinic system for the first time in Japan. For patients with NF1, which is a hereditary and systemic disease associated with a high incidence of malignant tumors, it will be of great benefit when the number of such clinics in Japan and the rest of Asia is increased.

## Introduction

Neurofibromatosis type-1 (NF1) is a hereditary disease that affects 1 in 2500–3000 people regardless of gender or ethnicity^1, 2^. Diagnosis is made with reference to the diagnostic criteria of the Japanese Dermatological Association, which were prepared based on the diagnostic criteria proposed by the National Institute of Health in 1988^3^. There is a remarkable phenotypic difference and an unpredictable lifelong course of the condition^4^. NF1 is characterized by the development of Café au lait plaques and benign neurofibromas^1^. Comorbidities that occur in adulthood include malignant tumors, central nervous system tumors, angiopathy, and cognitive impairment^1, 5–7^. Up to 60% of NF1 patients develop plexiform neurofibromas, which can cause pain, disfigurement, and malignant changes^8^. Malignant peripheral nerve sheath tumor (MPNST) is the most common and serious malignant tumor based on NF1, and the risk of developing it in a lifetime is reported to be 5.9–15.8%^9–11^. Patients with NF1 have been reported to develop malignancies at a 2.7–4 times higher risk than the general population^12–14^, and life expectancy is reduced by 8–20 years^15, 16^.

Thus, for NF1 patients who have various signs/symptoms and a high incidence of malignant tumors that have a great influence on the life prognosis, it is inappropriate to treat them in a single department. In the United States, the Neurofibromatosis Clinic Network was established by the Children's Tumor Foundation in 2007 to standardize and raise the level of clinical care for neurofibromatosis nationwide and integrate research into clinical care practices (https://www.ctf.org/research/nf-clinic-network). There are now 63 accredited institutions located in 32 states, and each institution has a multidisciplinary approach to NF1 patients^17^. On the other hand, in Japan, there was no multidisciplinary NF1 clinic, and so NF1 medical treatment was performed only by a specialist in a single department generally for one manifestation. There were many problems such as delays in medical treatment for other symptoms in NF1 caused by medical treatment in a single department. In order to overcome these problems, we have built and promoted an in-hospital NF1 medical care network with multiple departments and specialties since 2014. The purpose of this study is to introduce the process of launching this NF1 medical care network and its progress.

## Materials and methods

At our hospital, a working group for the establishment of the NF1 in-hospital medical care network was formed in 2013 by members of pediatrics, neurosurgery, orthopedics, and genetic counselors who have been involved in NF1 medical care. This working group discussed and decided the outlines of medical care network for NF1: departments in charge of medical treatment; flowchart of medical treatment by multiple departments; symptoms that should be noted in medical treatment for NF1 patients according to age. In this process, we had the cooperation of a pediatric/clinical geneticist instructor (S. M.) at the Aichi Developmental Disability Center as an advisor. With the consent of the chairman of each department involved in NF1 medical care, the NF1 in-hospital medical care network of Nagoya University Hospital was established. This network is managed by the director of the Genome Medical Center.

**Figure 1:**
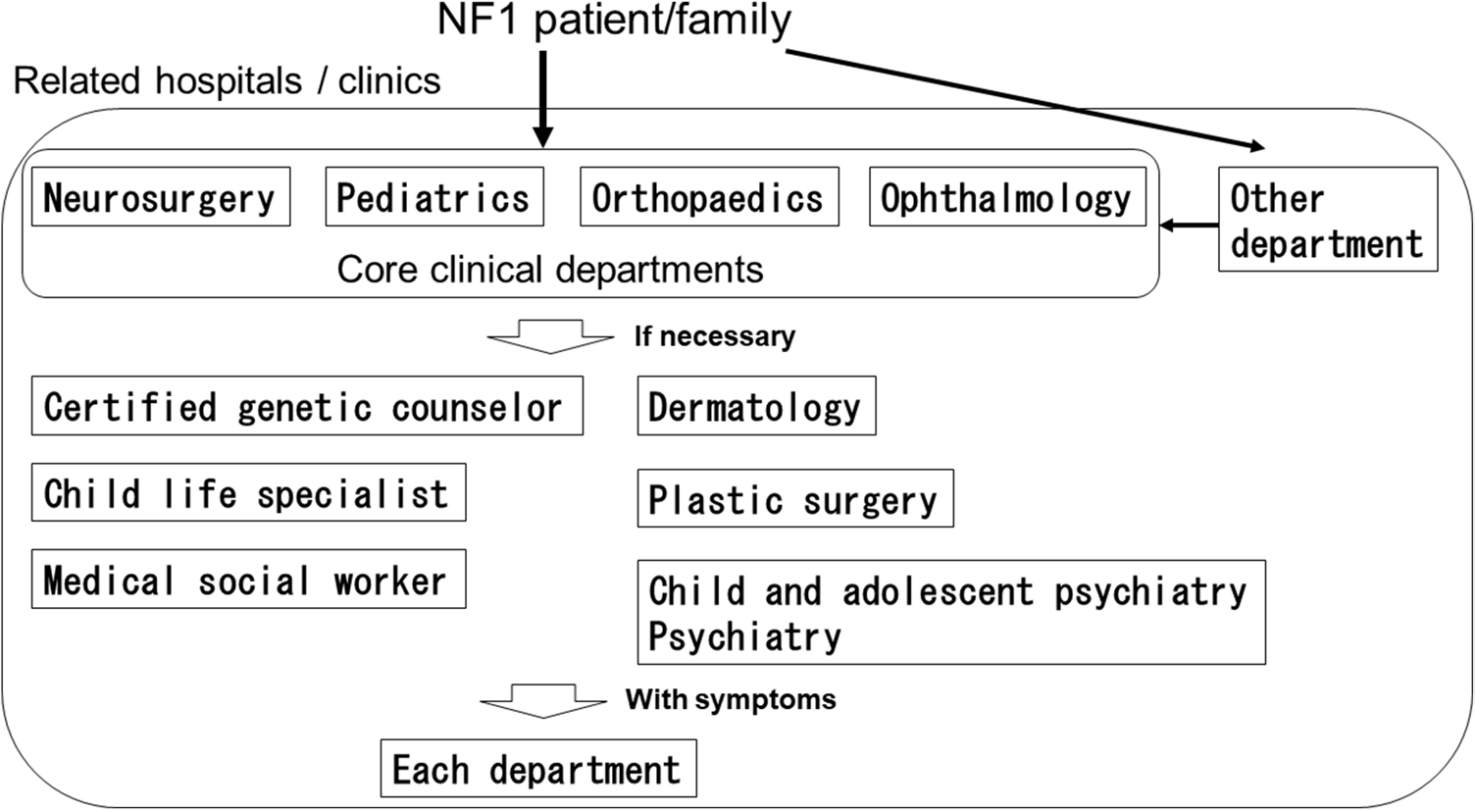
Medical treatment flow for NF1 patients under 15 years old in our institution.

In January 2014, we started multidisciplinary medical care through the NF1 in-hospital medical care network (Fig. 1). For example, if the patient is 15 years old or younger, pediatrics, ophthalmology, neurosurgery, orthopedics are regarded as essential departments, and the items to be treated in each department are evaluated (Table 1). If the patient/family wishes, genetic counseling will be provided by a certified genetic counselor. In addition to the four core departments, the network consists of dermatologists, plastic surgeons, psychiatrists, genetic counselors, child life specialists, and medical social workers (Table 2). We distribute pamphlets explaining this NF1 medical care network to related hospitals and clinics of our hospital and have them refer NF1 patients to our hospital.

**Table 1:** Items to be evaluated by each department in NF1 patients up to 15 years old.

Department	Mandatory or not	Items
Pediatrics	Mandatory	Physical/mental developmental retardation, high blood pressure, exophthalmos, sexual maturity
Ophthalmology	Mandatory	Sight, intraocular pressure, iris nodule, optic nerve glioma
Neurosurgery	Mandatory	Brain tumor, hydrocephalus, optic nerve glioma
Orthopedics	Mandatory	Scoliosis, limb dysplasia, deep-seated neurofibroma, MPNST
Dermatology, Plastic Surgery, Psychiatry, Otorhinolaryngology, Others	If patient/family wish	Skin lesions, cutaneous neurofibromatosis, diffuse neurofibromatosis, developmental disability, hearing, explanation/support to family members, etc

**Table 2:** Medical staff involved in NF1 medical care and related items.

Medical staff	
Genetic counselor	Consultation on heredity, patient/family support
Child life specialist	Partner of childhood, mental support before and after surgery
Medical social worker	Explanation of the medical insurance system, assistance for various economic, psychological and social problems

Since the NF1 in-hospital medical care network was launched in January 2014, medical records of NF1 patients who have been seen in this network have been prospectively created. Orthopedic oncologists (Y.N., K.I.) have been in charge of preparing these medical records. The contents are the date of the first visit to our hospital, age at the time of the first visit, gender, family history (including whether familial NF1 or non-familial NF1 is present), the department in which the examination is to be performed and NF1-related signs/symptoms in each department. Based on this record, we investigated the progress of this in-hospital NF1 medical care network since its start. We also conducted an epidemiological survey of NF1-related symptoms in orthopedics from the medical records. This practice and research was conducted in accordance with the principles set out in the Declaration of Helsinki.

### Ethics approval and consent to participate

Ethical approval was given by the institutional review board at the Nagoya University. The need for informed consent was waived by the retrospective design of the study based on anonym data.

## Results

In January 2014, we started the NF1 in-hospital medical care network. For NF1 patients who had their first visit before then and had been continuously treated, we requested each department to provide medical care as an NF1 network. Therefore, NF1 patients whose first visit date was before 2014 are also included in the medical records of NF1 network.

**Figure 2:**
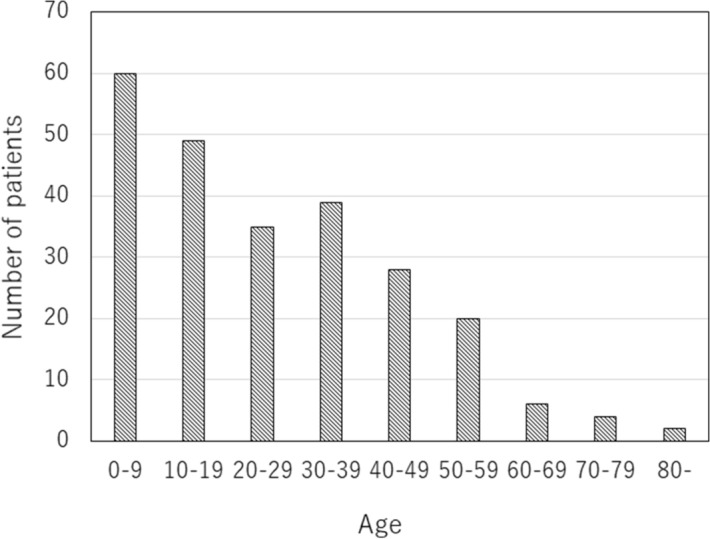
Number of NF1 patients by age group at first visit.

From January 2014 to December 2020, 246 patients were enrolled in the NF1 patient list and medical records. Excluding the three patients whose age at the first visit was unknown, mean age was 26.0 years ranging from 3 months to 80 years. The younger the age at the first visit, the higher the patient number distribution (Fig. 2).

**Figure 3:**
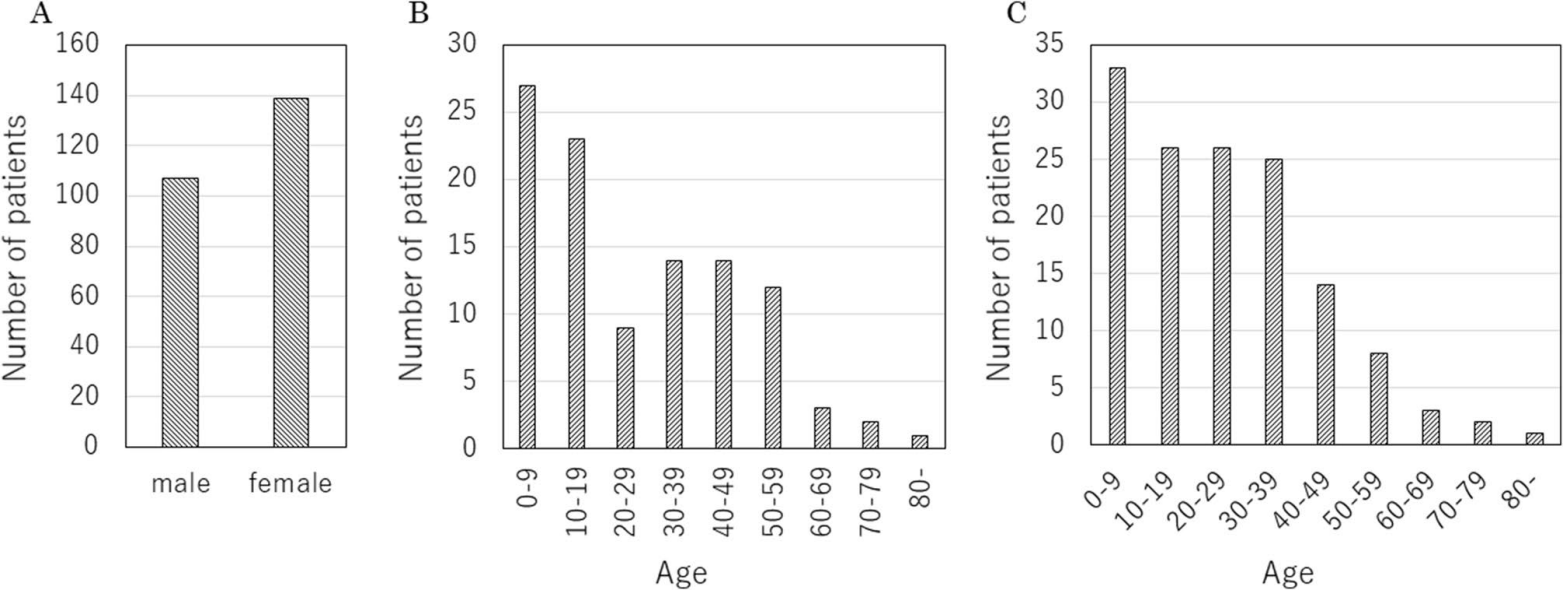
Number of NF1 patients by gender. (**A**) total, (**B**) male by age group at first visit, (**C**) females by age group at first visit.

There were 107 males (41%) and 139 females. Looking at the number of first-time patients by age group by gender, the number of males in their 20 s and 30 s was markedly lower than that of females (Fig. 3).

**Figure 4:**
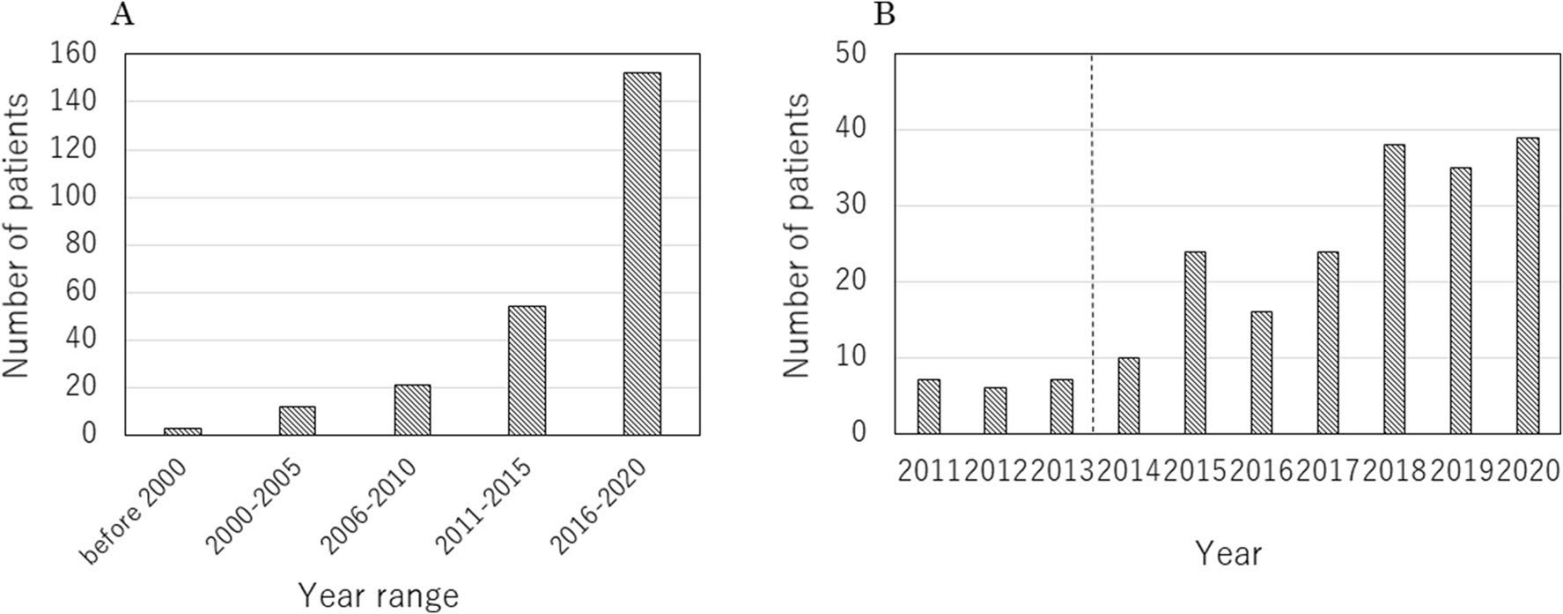
Number of NF1 patients by year. (**A**) year range separated by 5 years. (**B**) recent years. The dotted line indicates the time when the NF1 in-hospital medical care network was started.

Divided by 5 years, the number of first-time NF1 patients is increasing markedly. Especially after 2011, the number of patients has increased since the year when the medical care network was started (Fig. 4). Excluding 15 patients whose family history is unknown, familial NF1 was 101 (44%), and non-familial was 130, with non-familial slightly predominant.

**Figure 5:**
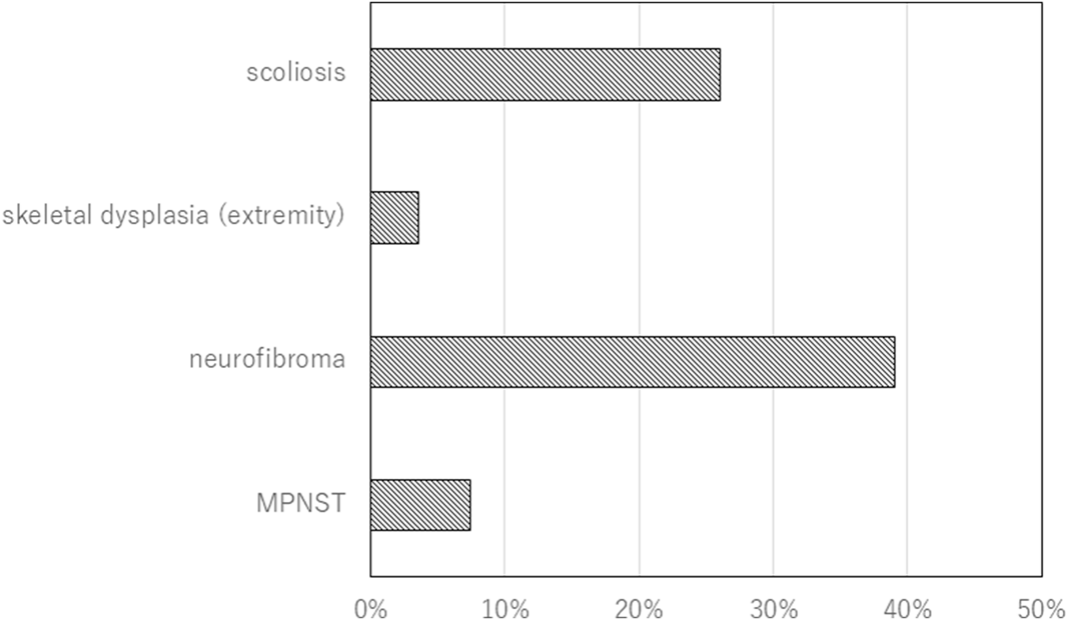
Diseases treated by orthopedic surgeons. Ratio to the total number of patients. Neurofibroma: deep-seated neurofibroma counted. Cutaneous neurofibroma not counted.

Regarding orthopedic signs, there was a description that was investigated in 230 patients. Scoliosis was present in 60 cases (26%), and bone abnormalities in the upper arm, forearm, and tibia were observed in 8 cases (3.5%). Neurofibromas other than cutaneous ones were found in 90 cases (39%) and MPNST in 17 cases (7.4%) (Fig. 5).

## Discussion

There have been no reports in the English literature of multidisciplinary NF1 clinics conducting medical care in Asia including Japan. It is often difficult for NF1 patients to be treated in many departments each time because of the hospital system or the staff who can handle them. There are several reports of NF1 clinics in Western countries, especially pediatric NF1 clinics. A report from Northern Ireland described the treatment of NF1 patients under the age of 16 years with an average of 4.6 years^18^. In Australia, the average age at consultation is 5 years and the male–female ratio is 1.05:1^19^.

Setting up an NF1 clinic for adults is more difficult than for children. In Australia, at the age of 18, individuals with NF1 must be transitioned to the adult health care system, for most adults with NF1 patients being treated by general practitioners who are in charge of referrals to specialists. However, most general medical practitioners have no training in the management of NF1. In a recent Australian study, most adult NF1 patients have not undergone regular disease monitoring since the transition from pediatrics to adulthood, and importantly, some adult NF1 patients developed new complications in adulthood, such as spinal-related medical conditions and chronic pain^20^. There was a report of an adult NF1 clinic in Canada, where the average age of patients was 32.6 years^21^. This report states that among adult NF1 patients, only 9% were referred from pediatric centers that were seeing NF1, suggesting the difficulty in transition to adult NF1 care. This study also found that there are six other adult NF1 clinics worldwide reported, two in the United States^22, 23^, 2 Australian facilities^19, 24^, 1 Brazilian facility^25^, and 1 French facility^26^. Of these, only one facility sees only adult NF1 patients^23^, while the others treat adults together with children. In the present study, the transition was considered to be successful in our hospital, as almost all patients could continue to be treated by the multidisciplinary team excluding pediatricians after the age of 16. However, it seems that the reason why the number of patients up to teenagers is large in our hospital might be that parents take the initiative to send their NF1 children to our NF1 clinic. The decrease in the number of NF1 patients over the age of 20 years may be a problem in Adolescence and Young Adult generation^27^. In addition, the decrease in the number of male NF1 patients between the ages of 20 and 39 is considered to affect the difference in the number of male and female NF1 patients in the present study.

Even if an adult consults the NF1 clinic, it is important to decide how intensely he/she needs to be treated. Due to the wide clinical spectrum expressed in NF1 patients, it is difficult to determine appropriate levels for adult monitoring and the infrequent occurrence of most complications^28^. Age-specific monitoring of symptoms and education of patients are important in managing NF1 patients. Adults with severe or complex symptoms need to attend an NF1 clinic, but adult NF1 patients with only mild symptoms need to know when to seek medical advice^29^. Symptoms such as rapid expansion of existing tumors, chronic pain, and neurological deficits require immediate medical attention and prompt examination and treatment^5, 13, 20, 29^. Personal education and support in self-management is important in the care of long-term NF1-related disorders^30^. An important matter in self-care is to activate the consciousness of the patient^31^, which means that NF1 individuals are ready to take action to manage their healthcare^32^. Increasing the level of interest improves health-related outcomes^31, 33^. Annual clinical surveillance is recommended for all NF1 individuals, however, there is evidence that some adults do not comply with these guidelines^7, 20^. Taken all together, in our hospital, we explain the importance of regular visits to patients with adult NF1 and have them understand the signs of suspected development of MPNST. We believe that it is important for them to be aware of such symptoms and to have them visit our hospital as soon as they notice them.

NF1 has been selected as one of 333 designated intractable diseases in Japan (Designated intractable disease No. 34) (https://www.nanbyou.or.jp/entry/3991). When the severity exceeds a certain level, the specified medical expenses subsidy system is applied based on the Intractable Diseases Law in Japan. However, many doctors are not familiar with the designated intractable disease system, and so some NF1 patients do not receive medical assistance appropriately. In that sense, it is also important to consolidate NF1 patients in our hospital and give them the opportunity to benefit from medical assistance appropriately. Since registry system has not been completely established in Japan yet, it is unknown how many NF1 patients there actually are. If the efforts of NF1 clinics like our hospital spread all over Japan and NF1 clinics can be established in each region, with the establishment of a registry system, accurate epidemiological data of NF1 patients will become available in the future.

The COVID-19 pandemic has greatly affected the medical field. NF1 clinical care in the United States has also been greatly affected. A study by Radtke et al. indicated that about two thirds of the NF1 clinics reported a greater than 50% decrease in the number of available patient appointments, and modified clinical surveillance and research protocols^34^. In Japan as well, the decrease in the number of examinees delays the detection of disease, and results in a decrease in medical income. However, even under such circumstances, the number of patients who visited our in-hospital NF1 clinic increased in 2020. This may be because of the strong interest in this first such NF1 clinic in Japan and differences in the influence of the pandemic between the United States and Japan.

As an initiative of All Japan, the Japanese Society of Recklinghausen Disease has set up a medical care network at multiple facilities in Japan with the aim of consolidating NF1 medical care (http://plaza.umin.ac.jp/~jsrd/network/index.html). In addition, the society is actively conducting academic activities with the aim of equalizing NF1 medical care in Japan. These are very meaningful activities for NF1 patients and their families. However, since the number of doctors and facilities that can specialize in NF1 medical care listed on the academic society network is still small, patients and their families may need to travel long distances to receive medical treatment from these specialists. It is necessary to increase the number of NF1 clinics in Japan so that comprehensive NF1 medical care can be performed in a single facility like our hospital.

There are several limitations in our report. First of all, it may not be possible to extract all the information from the medical record, and the incidence of each disease (scoliosis, deep-seated neurofibromatosis, etc.) may be higher. Second, because the orthopedic oncologist has created a list and a record of NF1 patients who have been treated on the medical care network, it is possible that NF1 patients who have not been treated by an orthopedic oncologist are missing from the list. However, we request that all NF1 patients treated in related departments be referred to orthopedics. Third, the data may contain a selection bias, as initially severely symptomatic NF1 patients were referred to this NF1 clinic. However, recently, the number of patients, who are referred when they are diagnosed with NF1 or when Café au lait spots are observed at birth, is increasing, and so it may become like population based data in the future. Fourth, in this research, we cannot provide scientific information at present. However, in the future, it will be possible to use this registry to accumulate data such as whole body MRI that is prospectively imaged to detect deep-seated plexiform neurofibroma, and send out scientific data. Finally, this clinical care does not include nurses that specialize in NF1 practice, and will be necessary in the near future.

## Conclusions

We have launched what is probably the first multidisciplinary NF1 clinic system in Asia. For patients with NF1, which is a hereditary and systemic disease with a high incidence of malignant tumors, it would be a great relief to increase the number of such clinics in Japan and the rest of Asia.

## Data Availability

The datasets supporting the conclusions of this article are available from the corresponding author (YN) on reasonable request.

## Abbreviations

NF1: Neurofibromatosis type 1
MPNST: Malignant peripheral nerve sheath tumor
